# High Risk for Persistent Peri-Prosthetic Infection and Amputation in Mega-Prosthesis Reconstruction

**DOI:** 10.3390/jcm12103575

**Published:** 2023-05-20

**Authors:** Christina Berger, Catharina Parai, Jonatan Tillander, Peter Bergh, David Wennergren, Helena Brisby

**Affiliations:** 1 Institution of Clinical Sciences, Department of Orthopaedics, Sahlgrenska Academy, University of Gothenburg, 416 45 Gothenburg, Sweden; 2 Department of Orthopedics, Sahlgrenska University Hospital, 413 45 Gothenburg, Sweden; 3 Institution of Biomedicine, Department of Infectious Diseases, University of Gothenburg, 413 90 Gothenburg, Sweden

**Keywords:** peri-prosthetic joint infection, mega-prostheses, bone reconstruction

## Abstract

A peri-prosthetic joint infection is a feared complication after mega-prosthesis reconstruction of large bone defects. The current study investigates how patients operated with a mega-prosthesis due to sarcoma, metastasis, or trauma, are affected by a deep infection focusing on re-operations, risk for persistent infection, arthrodesis, or subsequent amputation. Time to infection, causative bacterial strains, mode of treatment and length of hospital stay are also reported. A total of 114 patients with 116 prostheses were evaluated, a median of 7.6 years (range 3.8–13.7) after surgery, of which 35 (30%) were re-operated due to a peri-prosthetic infection. Of the infected patients, the prosthesis was still in place in 51%, 37% were amputated, and 9% had an arthrodesis. The infection was persistent in 26% of the infected patients at follow-up. The mean total length of hospital stay was 68 (median 60) days and the mean number of reoperations was 8.9 (median 6.0). The mean length of antibiotic treatment was 340 days (median 183). Coagulase-negative staphylococci and *Staphylococcus aureus* were the most frequent bacterial agents isolated in deep cultures. No MRSA- or ESBL-producing *Enterobacterales* were found but vancomycin-resistant *Enterococcus faecium* was isolated in one patient. In summary, there is a high risk for peri-prosthetic infection in mega-prostheses, resulting in persistent infection or amputation relatively often.

## 1. Introduction

Reconstruction of large segmental bone defects with a mega-prosthesis is still a rare procedure, although the indications have been extended from exclusively sarcoma patients to major traumatic skeletal/joint injuries, methastases, and sometimes to patients experiencing failure of conventional endoprostheses. Modular mega-prostheses are available for all large joints such as the shoulder, knee and hip, and their adjacent skeletal segments. This method is considered safe and has demonstrated good functional results, but the frequency of complications, i.e., infections and mechanical failure, is higher than for conventional endoprostheses [1,2,3,4,5]. There are several plausible explanations for the high number of complications. A larger amount of metal, decreased quality, and less amount of soft tissue, and—in oncologic patients—subsequent administration of adjuvant chemotherapy or radiation treatment, are all providing poor prerequisites for uncomplicated healing [2].

One of the most feared complications is peri-prosthetic infection, often resulting in repeated surgery, prolonged antibiotic treatment, and sometimes even in an amputation of the affected limb. Coagulase-negative staphylococci has been reported to be the most common bacteria, and may be difficult to treat due to a combination of extensive antimicrobial resistance, biofilm forming propensity, and delayed diagnosis [6].

The frequency of postoperative infections, and their effect on outcomes after mega-prosthesis treatment, in tumor as well as in other patient groups, highly varies [7,8,9,10]. Sometimes it is difficult to differentiate infection from aseptic complications and determine the causative organism in a peri-prosthetic infection [11]. A postoperative peri-prosthetic infection that is not eradicated may cause a chronic infection resulting in life-long antibiotic medication or amputation of the affected limb.

The aim of this study was to evaluate the incidence and the time to peri-prosthetic infection after index mega-prosthesis surgery, to identify the causative bacteria, and the consequences especially in terms of reoperations, persistent infections and amputations in a Nordic centre. Furthermore, data of other medical procedures that may have been linked to the peri-prosthetic infections were explored.

## 2. Patients and Methods

This retrospective observational study included all patients undergoing primary mega-prosthesis reconstruction at any body location at the Sahlgrenska University Hospital (Gothenburg, Sweden) between January 2006 and May 2019. Data were collected until June 2021. Complications other than infections as well as a detailed description of baseline characteristics and a brief description on peri-prosthetic infections for the present cohort have been included in a previous paper, focusing on the overall risk of adverse events. Frequency and type of complication and the overall risk for amputation for reconstructions of different joints were reported [12].

All patients treated with a mega-prosthesis during the study period were identified using the hospital’s surgical planning system, followed by a review of their medical records. Information on mega-prosthesis location, number and reason(s) for reoperations, type and length of prophylactic antibiotics, local antibiotics administrered at surgery, and time from surgery to first eventual infection was extracted. The diagnosis of peri-prosthetic infection was for each patients set by the the treating clinician, the information in the charts for all patients defined as having a peri-prosthetic infections were retrospectically reviewed and were found to fullfill the European Bone and Joint Infection Society’s (EBJIS) definition of a peri-prosthetic infection [13]. Records of bacteria isolated in deep cultures from the first reoperation and the total number of days for patient hospital admission due to mega-prosthesis infection were also extracted. The antibiotic therapy given for the infections consisted of a variety of different antibiotics, based on resistance patterns after recommendations of a senior consultant in infectious medicine (data not explored in the present article).

Further, at the end of the observational period, the current status of each prosthesis was noted, i.e., whether the original prosthesis was still implanted without revision of parts, revised and still implanted, removed plus arthrodesis performed, or if an amputation was executed. Data on resolved infection was noted as eradicated (defined as no remaining signs of infection at last visit and CRP < 5 after removal of antibiotics for at least three months) or still present (defined as chronic draining sinus and/or life-long suppressive antibiotic treatment) were also extracted.

The study was approved by the Swedish Ethical Review Authority, Gothenburg 2019-04041.

The mega-prostheses primarily used in this study was the Modular Universal Tumor and Revision System, MUTARS™ (Implant Cast, Buxtehude, Germany). As the name indicates, this implant system is modular, allowing adjustments and adaptations according to different skeletal segments/joint resections as well as to individual factors. The mega-prostheses in the present study were used for reconstructions of knee, hip, pelvis, shoulder, and elbow joints and subsequent parts of long bones, as previously described [12]. Antibiotic-loaded bone cement were used for all cemented prostheses.

All patients were routinely given intravenous antibiotics perioperatively with the first dose 30–60 min before the start of the surgery, and thereafter every 6–8 h during the first 24 h. Extra doses were administred when the surgical time exceeded three hours. The primary choice was Cefuroxim, 1.5 g/dose and—in case of penicillin allergy—Clindamycin, 600 mg/dose, were given. From 2015, local antibiotics were administrered subfascially in the wound perioperatively on most patients at the primary surgery. The products used were Cerament^®^ V, or Cerament^®^ G, (Bonesupport™, Lund, Sweden). Cerament^®^ is a synthetic bone substitute consisting of 40% hydroxyapatite and 60% calcium sulphate with the addition of vancomycin or gentamycin providing a high local concentration of antibiotics. The prosthesis was dressed in Cerament^®^ before the fascia was closed over the prosthesis.

### Statistics

Descriptive statistics are presented as percentage, median and range or mean, and SD, where relevant. Mann–Whitney U-tests for non-parametric independent groups were used for comparing means between infected and non-infected groups regarding the number of reoperations (regardless of reason). A Kaplan–Meier curve was created for implant survival for infected respective non-infected patients. Possible correlations between the number of reoperatins in infected patients and having an amputation were performed with Point-Biserial correlation tests. All statistical analyses were performed using IBM SPSS version 27.

## 3. Results

### 3.1. Demographics and Overview of Diagnosed Peri-Prosthetic Infections

**Figure 1:**
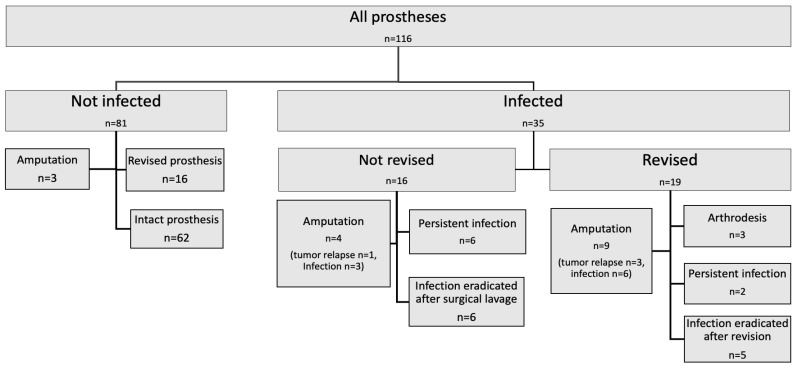
Outcome for infected and not infected prosthesis after surgical and medical treatment.

In total, 114 patients (54 females (48.2%)) were provided with 116 mega-prostheses. Every patient was followed until the end of the study period (June 2021) or until death. The median age at surgery was 60.4 years (range 16–78) and the median follow-up time was 7.6 years (range 3.8–13.7). The indication for surgery was bone or soft tissue sarcoma in 53.5% of the patients, metastases of other malignancies in 36.0% and benign tumours, fractures, and/or revision of conventional knee- or hip- prosthesis in 10.5%. A peri-prosthetic infection was diagnosed in 35/114 of the patients (30%) by the treating clinician. All these 35 patients fulfilled the EBJIS criteria for a confirmed peri-prosthetic infection, in 32/35 at least 2 positive samples with the same microorganism were found (sometimes together with other confirmative signs) and in the three patients without any positive cultures the criteria “sinus tract with evidence of communication to the joint or visualization of the prosthesis” was fulfilled. The infection rates for prosthesis in the upper extremities were 25% (5/20) and for the lower extremity prosthesis 31% (30/96). All infections were treated with antibiotics in combination with a complete or partial prosthesis revision in 19/35 patients or with surgical lavage without revision of the prosthesis in 16/35 patients (Figure 1).

Among those 35 diagnosed with a peri-prosthetic infections, 21 were men and 14 were women. In 5/21 (24%) of the men and 4/14 (29%) of the women with an infection, the end result became amputation.

**Figure 2:**
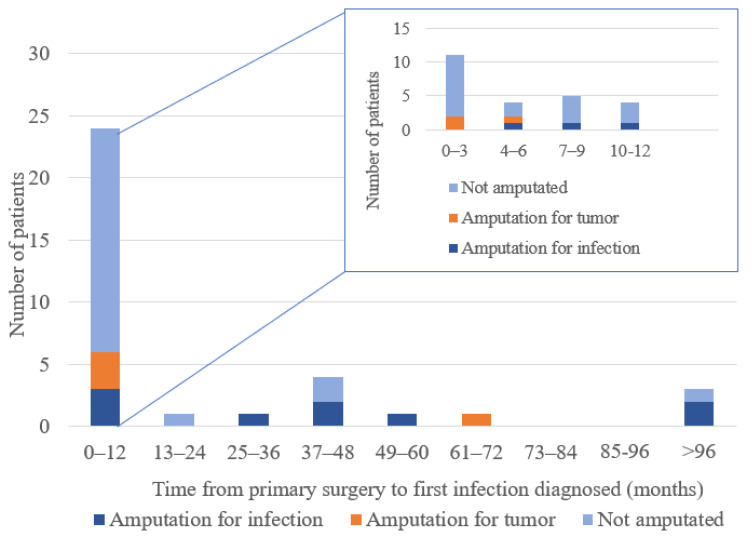
Time from primary surgery to first infection diagnosed (months). Two thirds (n = 24) of all infections (n = 35) were observed within one year after primary surgery.

The majority of the infections were diagnosed during the first 12 postoperative months (24/35 or 69%) and 11 of these (31%) within the first 3 months. (Figure 2). Peri-prosthetic infections within the first year without any other diagnosed infection or underlying event were regarded as caused by contamination during the index surgery. This was the case in 18/35 (43%) of the patients. In 14/35 patients, the peri-prosthetic infection was suspected to be caused by a medical intervention other than the primary surgery, or from a distal infection; (i) infection of a central venous catheter used for chemotherapy (n = 4), (ii) reoperation for a peri-prosthetic complication other than infection (n = 6), (iii) a superficial wound infection in another body location (n = 3), and (iv) a tooth infection (n = 1) (Table 1). In 3/35 patients, the mode of pathogen transmission was not possible to determine/speculate on.

### 3.2. Type of Detected Bacteria

As shown in Table 1*, Staphylococcus* species were the most common bacteria. Of the 35 infected patients, 25 had coagulase-negative staphylococci in their cultures. *S. aureus* was the second most common isolate (11/35) *Streptococcus* and *Enterococcus* spp. were identified in 12/35 patients, among which amoxicillin-sensitive *E. faecalis* was identified in 7 patients, ß-hemolytic streptococci Group A in 2, Group G in 1, and *Streptococcus mitis* in 1. One patient had a positive culture for *E. faecium* (vancomycin-resistant enterococcus (VRE). *Candida albicans* was found in two patients and *Bacillus cereus*, *Pseudomonas aeruginosa*, anaerobes, and other bacteria were found in single cultures in seven patients. Cultures from three patients with clinically overt infections were negative.

### 3.3. Single Bacterial Species or Multiple Species

In 14/35 of the infected patients (40%), a single bacterial pathogen was identified. In the remaining patients, a mixture of several bacterial species were found. Of the identified single strain infections, nine were coagulase-negative staphylococci—infections, three *S. aureus,* one *Actinomyces europeus,* and one *Cutibacterium acnes*.

In patients with multiple pathogen isolates (60% of the infected patients), the most common combinations of bacteria were coagulase-negative staphylococci and *S. aureus* (7/18), and coagulase-negative staphylococci and *E. faecalis* (4/18).

### 3.4. Onset of Infection Related to Bacterial Species

Infections caused by a single pathogen were found in 50% (12/24) of the patients diagnosed during the first postoperative year and in 18% (2/11) of those that were diagnosed later than one year after the index surgery. No such pattern was seen in patients in whom several pathogens were isolated. Half of the patients with a mixed infection (9/18) were diagnosed during the first postoperative year, and the others at a later stage.

### 3.5. Bacterial Strains in Relation to Clinical Consequences

At the end of the observational period, out of 14 cases with a single pathogen infection, six were considered free from infection with a remaning prosthesis, four were amputated (three due to tumor, one due to infection), one had an arthrodesis, two had a persistent infection, and one patient had died because of the malignancy just a short time after the infection was established.

Out of the 18 patients that presented with multiple pathogen isolates, eight were amputated (all but one due to the infection) and four died shortly after the index surgery from the malignancy. The only patient with a mixed infection who kept a functional prosthesis without signs of infection, had an early wound rupture but healed after a single reoperation.

The eight patients with a persistent infection at follow-up/until dead were all infected with coagulase-negative staphylococci, either as the single pathogen or in combination with other bacteria.

### 3.6. Antibiotic Prophylaxis and Antibiotic Therapy

All but three patients received cefuroxim as antibiotic prophylaxis at the time of the index surgery and the remaining three received clindamycin because of allergies. Two out of three of the patients given prophylaxes with clindamycin got a peri-prosthetic infection. Of the patients that received cefuroxim, 33/111 were diagnosed with a peri-prosthetic infection.

In 31% of the patients (31/99) not receiving local antibiotics (gentamycin or vancomycin) during the primary surgery and in 4/15 (27%) of patients given local antibiotics, a deep infection was diagnosed. Two patients recieved vancomycin; none of those patients got infected, the rest of the fifteen received gentamycin.

The median time of antibiotic treatment was 183 days (range 5–2216). The patient with the shortest antibiotic administration, only five days, was clearly an outlier and the short treatment was the result of a severe infection in a prosthesis involving the knee joint, which is why he underwent an acute amputation.

### 3.7. Length of Stay in Hospital and Number of Reoperations

The mean total length of hospital stay for patients treated for a peri-prosthetic infection was 67.6 days, (range 19–236 days). The number of reoperations was median 6.0 (range 1–51 reoperations) for infected patients and a median of 1.5 (range, 1–4, *p* < 0.001) reoperations for non-infected patients.

### 3.8. Effect of Infections on Prosthesis Survival and Amputations

**Figure 3:**
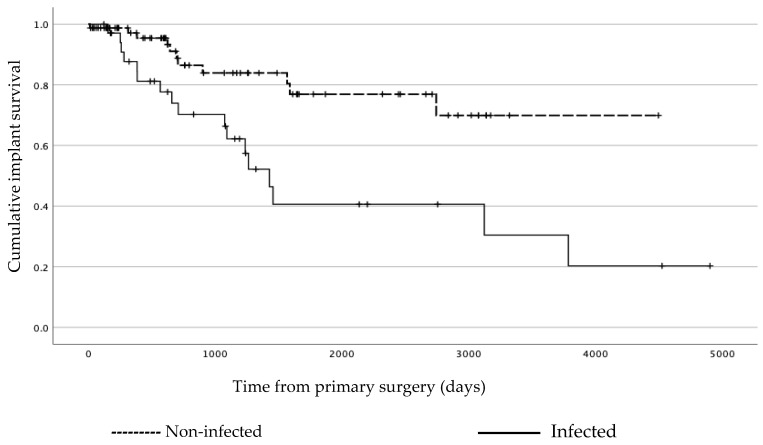
Kaplan–Meier analysis showing implant survival for non-infected and infected patients, respectively. In the analysis, any cause for exchanging the prosthesis or amputation were set as events of interest. Relaps of the tumor or death of the patient were censored.

At the end of the study period or until death, 51% (18/35) of patients with infection had a retained or a revised prosthesis. In the group of non-infected patients, 96% (78/81) had kept their original prosthesis or had undergone a revision for complications other than infection. For prosthesis survival in the infected and non-infected patients, see Figure 3.

Amputation was performed in 13 (37%) of the infected patients, of which 9 were caused by the infection and 4 by tumor relapse. There were no other reasons for amputations than tumor relapse or infections. In the non-infected patients, 3/81 (4%) had an amputation during the study period, all due to tumor relapse, see Figure 1. All amputations due to infection were performed on the lower extremities, eight transfemoral amputations and one exarticulation of the hip. Three infected patients had a secondary arthrodesis, one had the prosthesis removed with no new reconstruction, and one was lost to follow-up at one year after the primarysurgery. In the non-infected group, 4% (3/8) were amputated, all due to tumor relapse. None were treated with arthrodesis (Figure 1).

### 3.9. Surgical Treatments of Infections Related to Clinical Outcome

In 16 patients, lavage without revision was the first choice of treatment. Half of these patients (n = 8) were considered as having an eradicated infection at the end of the study. The remaining 19 infected patients had a complete or partial prosthesis revision. Six of them had no signs of infection at the end of the observational period (Figure 1).

### 3.10. Reoperations in Relation to Amputations

**Figure 4:**
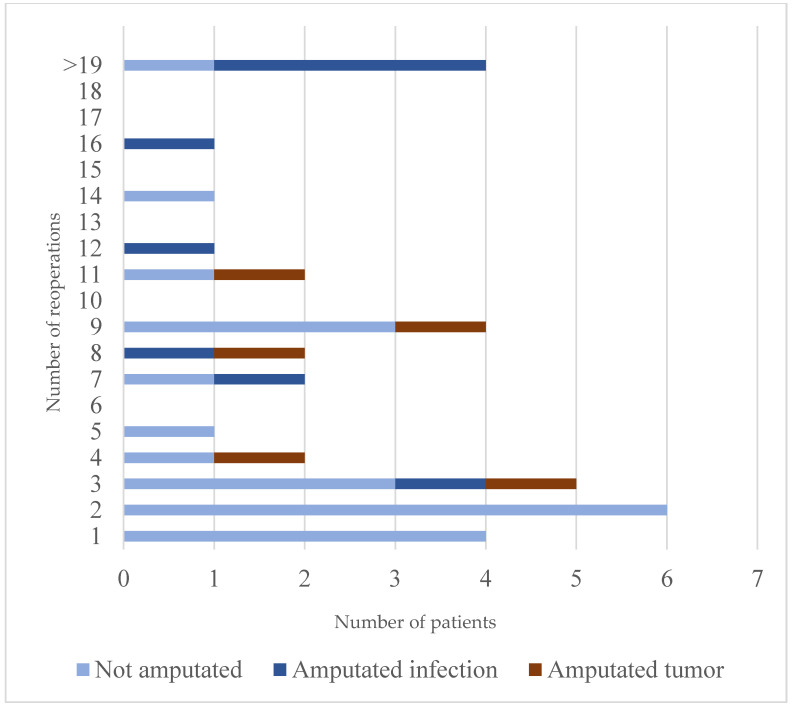
Number of reoperations including amputation for sarcoma patients and for patients reconstructed for other causes diagnosed with peri-prosthetic infection. Three amputated patients had another diagnosis than sarcoma as an indication for the prosthesis reconstruction and were all amputated due to infection.

In this material, the risk for amputation appeared to increase with the number of reoperations. When patients had five or more reoperations, the risk for amputation was relatively high, 7 out of 17 patients. This is in comparison with the 3 out of 18 patients reoperated less than 5 times that were amputated (Figure 4). Point–Biserial correlation analysis showed a significant correlation between the number of reoperations and amputation in infected patients (r = 0.524, *p* < 0.001).

**Figure 5:**
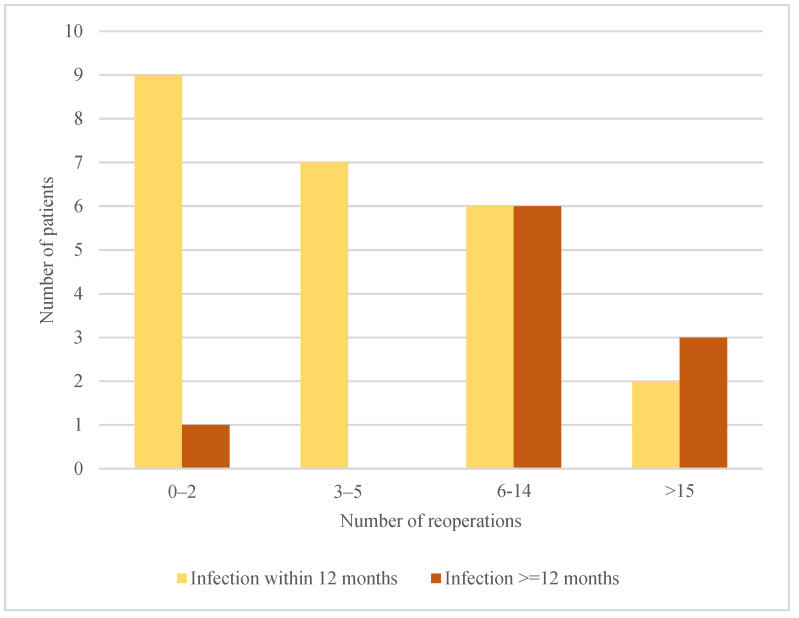
Time to first infection and number of reoperations.

Furthermore, as shown in Figure 5, when the infection was diagnosed later than twelve months after the index surgery, the number of reoperations needed before the infection was eradicated was higher compared to infections diagnosed 0–12 months after index surgery.

## 4. Discussion

In the current study, 35/114 of the patients with a mega-prosthesis suffered a peri-prosthetic infection. Frequent reoperations, weeks or even months in hospital, and long-term antibiotic therapy appears to be what awaits every third mega-prosthesis patient. The risk for long-term effects, e.g., a persistent infection requiring life-long antibiotics or amputation of the affected limb, are high when receiving an infection in a limb treated with a mega-prosthesis.

In comparison with other studies on mega-prostheses, as well as all types of endoprosthesis in sarcoma patients, the present study demonstrated a higher proportion of peri-prosthetic infections. In a recent paper by Khakzad et al. on mega-prosthesis, an infection rate in the knee region of 28% was reported, which is similar to the overall infection frequency in the present study [14]. In our study, mega-prostheses in the knee region accounted for only about 40%. Further, the proportion of primary bone tumors, known to be a negative predictive factor for infection, was in the same range in our study as in the study by Khakzad et al., 53% vs. 57%. In a review on endoprostheses used in sarcoma patients by Racano et al., the infection rate was reported to vary from 0 to 25%, with an overall calculated risk of infection of about 10% [8]. The higher rate of infections in our series can only be speculated upon, but may be attributed to differences in patient characteristics and the relatively long follow-up period. Other factors that may also be influential are the type of tumor, type of oncological treatment, and the timing of surgery in relation to this treatment, as well as the choice and timing of antibiotic prophylaxis.

The clinical outcome for patients with a peri-prosthetic infection was, as expected, worse than for non-infected patients. The risk for amputation or chronic peri-prosthetic infection was considerably higher for patients with mega-prosthesis than for those with conventional endoprostheses [15,16]. In our study, it appeared that the larger the number of reoperations, the greater the risk for ending up with an amputation, suggesting that amputation as a treatment option maybe should be discussed with the patients earlier within the treatment period of an infection.

The high risk for amputation is partly caused by the difficulty to exchange the prosthesis for a new one, or a spacer, often used for conventional prosthesis, because of the large bone segment missing after a tumor resection. The present study indicates that infections occurring later than one year after the index surgery are more likely to result in an amputation or a chronic infection than those diagnosed during the first postoperative year. Further, patients that require multiple reoperations due to an infection have a high risk of ending up having an amputation.

The spectrum of bacterial species found were mostly in line with previous studies, with a large number of infections involving staphylococcus and streptocuccus strands. Similar proportions of staphylucoccus aureus infections were found in patients with infections diagnosed at 0–3 months, 3–12 months, or later than 12 months after receiving their prosthesis, which is similar to previously reported results [17]. In our patient cohort, many patients had ongoing medical treatments, e.g., cytostatic treatments, and thereby longstanding CVC’s, which together with infections in other parts of the body, may have contributed to this finding by hematogenous spread of bacteria. *Cutibacterium acnes* was detected in only 1/35 peri-prosthetic infections, which was an unexpectedly low number. Perhaps cefuroxim, which was routinely used as the first choice prophycactic antibiotic at our centre at the time, played a part in this low count. Further, only a few of the infections engaged a shoulder prosthesis, where *C. acnes* is more common than in lower extremity prostheses [18].

The low incidence of multidrug-resistant bacteria was a positive finding in the present study. The incidence of multi-resistant bacteria was much lower (1/35, representing 3%) than in previous reports [11,19,20]. For example, Zajons et al. reported a 26% rate of methicillin-resistant *S. aureus* [11]. The data in our study might justify the implementation of the same type of antibiotic prophylaxis for mega-prosthesis as for conventional prostheses for patients treated in Sweden.

In the present study, most patients had three doses of cefuroxim during the operating day. A longer time with antibiotic prophylaxis has been suggested to decrease the risk for surgical site infections. A North American study recently randomized patients receiving mega-prosthesis for bone tumors to get one or five days of antibiotic prophylaxis with cephalosporines, but no results are available as of yet [21]. In a systematic review, Thornley et al. could not show a decrease in surgical site infection after prolonged prophylaxis (i.e., longer than 24 h). However, this was in conventional hip- and knee-prothesis surgery [22]. It has to be taken into account that the incidence of peri-prosthetic infections in conventional joint prosthesis is much lower, between 0.5 and 2% [23,24]. One reason for the differences seen are that such surgeries are mainly performed on patients without any medical anti-tumoral treatment.

Notably, two of three patients in our cohort receiving clindamycin as prophylaxis got a peri-prosthetic infection, which may suggest this antibiotic to be less suitable than cefotaxime. The small numbers in our study, however, prevent conclusions, but a higher incidence in postoperative infections among patients treated with a knee-prosthesis for osteoarthritis with clindamycin as prophylaxis has been previously reported [25].

The antibiotic prophylaxis national guidelines used in our country has been based on the low overall incidence of multi-resistant bacteria within society and in hospitals [23]. On the other hand, some of the most feared bacterial species in orthopaedic surgery are the coagulase-negative staphylococci, often initially treated with parenteral vancomycin. It would, therefore, be of great interest to study the influence on infections, when using cephalosporin or cloxacillin and local vancomycin as prophylaxis in bone reconstructions with mega-prostheses. The combination of two or three antibiotics as prophylaxis has been suggested for a long time. However, the results in studies are still inconclusive [26].

The timing of the first dose of antibiotics has also been demonstrated to be of great importance [22,27]. Based on guidelines, the routine in our hospital is to administer the first dose at 30–60 min before the initiation of the surgical procedure. However, the retrospective nature of our data did not allow us to check if the routine was completely followed. Furthermore, the suspicion that other complications and/or medical interventions may have caused some of the peri-prosthetic infections support the need for prolonged or repeated antibiotic prophylaxis in relation to certain medical interventions for these patients.

The strength of the current study is that all but one patient was available for evaluation until dead or to the end of the follow-up period. A weakness of the study is the limited number of patients in the cohort, however, this number is similar to reports on this type of patients/procedure at other centers over the world [18].

## 5. Conclusions

A peri-prosthetic infection in patients with mega-prostheses is a common, and truly demanding condition, resulting in reoperations, long periods of hospitalization, and antibiotic treatment. The risks for persistent infection or amputation were relatively high in the present study, however, compared to reports from other countries, the incidence of bacterial strains with acquired antibiotic resistance was low. In summary, patients undergoing mega-prosthesis surgery should, due to the substantial risk of having an infection with concommitant need for additional treatments and the risk to end up with an amputation, be properly informed about the risks and effects of peri-prosthetic infections.

## Figures and Tables

**Table 1:** Most common bacterial agents, number of reoperations and adverse events associated with peri-prosthetic infection.

		Time to Peri-Prosthetic Infection Diagnosis (Months)		
		0–3 (n = 11)	4–12 (n = 13)	>12 (n = 11)
Three most common agents		CoNS ^1^ (n = 7) *S. aureus* (n = 2) *Ent. faecalis* (n = 2)	CoNS ^1^ (n = 9) *S. aureus* (n = 4) *Ent.faecalis* (n = 2)	CoNS ^1^ (n = 9) *S. aureus* (n = 5) *Candida* spp. (n = 3)
Age (y)		16–78 (median 54.5)	11–76 (median 45.0)	7–88 (median 55.0)
Female(n)		3/11	8/13	3/11
Sarcoma		8	8	7
No. of surgeries		1–8 (median 3)	1–51 (median 3)	1–25 (median 12)
Suspected associated events	CVC ^2^ infection	1	2	1
	Reoperation		2	4
	Other wound inf.		1	2
	Tooth infection			1

^1^ Coagulase-negative staphylococci, ^2^ Central venous catheter.

## Data Availability

The datasets used and/or analyzed during the current study are available from the corresponding author on reasonable request.
